# Vision-Threatening Ocular Adverse Events after Vaccination against Coronavirus Disease 2019

**DOI:** 10.3390/jcm11123318

**Published:** 2022-06-09

**Authors:** Mihyun Choi, Min-Hwan Seo, Kwang-Eon Choi, Sukyeon Lee, Boyoon Choi, Cheolmin Yun, Seong-Woo Kim, Yong Yeon Kim

**Affiliations:** 1 Department of Ophthalmology, Korea University Guro Hospital, Korea University Medicine, 148, Gurodong-ro, Guro-gu, Seoul 08308, Korea; mnyoung23@gmail.com (M.C.); perialseo4343@gmail.com (M.-H.S.); sukyeon56@hanmail.net (S.L.); eunice.b.choi@gmail.com (B.C.); 2 Department of Ophthalmology, Korea University Ansan Hospital, Korea University Medicine, 123, Jeokgeum-ro, Danwon-gu, Ansan-si 15355, Korea; kwangu2@hanmail.net (K.-E.C.); yuncheolmin@korea.ac.kr (C.Y.)

**Keywords:** vaccination, SARS-CoV-2, COVID-2019, ocular adverse events, vascular occlusions, uveitis, angle-closure glaucoma

## Abstract

A single-center retrospective observational case series was conducted. This case series enrolled patients who showed ophthalmic manifestations within one week after COVID-19 vaccination at Korea University Guro Hospital in Seoul, Korea, from May 2021 to January 2022. The medical records of patients who complained of ocular symptoms and showed ophthalmic adverse events within one week after COVID-19 vaccination were reviewed. Seventeen eyes from 16 patients with a mean age of 63.8 (range 33–83) years were included in the case series, and all symptoms developed within 1–7 days following inoculation. Retinal vein occlusion in nine eyes (52.9%), retinal artery occlusion in one eye (5.9%), newly developed anterior uveitis in one eye (5.9%), exacerbation of previously diagnosed panuveitis in two eyes (11.8%), and angle-closure attack with high intraocular pressure in four eyes (23.5%) were included. Twelve patients (75%) had been vaccinated with the AstraZeneca (AZD1222) and four (25%) with the Pfizer (BNT162b2) vaccines. Of these, 10 patients (62.5%) experienced ocular disease exacerbation after the first dose, 4 (25%) after the second dose, and 2 (12.5%) after the third dose (booster shot). Eleven patients (64.7%) underwent tests for hematological abnormalities, and three of them tested positive for anti-PF4 antibodies, but no abnormal findings were noted. A causal relationship between vaccination and the ocular manifestations could not be determined, which is a limitation of this study. However, clinicians should consider the effect of COVID-19 vaccination on ophthalmic disease. Further studies are required to elucidate the possible effects of COVID-19 vaccination on the eye.

## 1. Introduction

On 13 May 2021, the Royal College of Ophthalmologists in the United Kingdom issued a safety alert for retinal vein occlusion (RVO) in the immediate period (28 days) after vaccination for coronavirus disease 2019 (COVID-19) [1]. Vaccination against COVID-19 is now being conducted worldwide. In Korea, COVID-19 vaccinations began in March 2021, first among health care workers and vulnerable members of the community in March and April, and then expanded to all individuals older than 60 years of age in May and June. The AstraZeneca (AZD1222) and Pfizer (BNT162b2) vaccines are the two mainstays of the government-driven, nation-scale vaccination program initiated in South Korea. Approximately 20 million people were vaccinated as of August 2021, with those who received the AZD1222 and BNT162b2 vaccines accounting for 52% and 38% of the recipients, respectively. To date, 43 million people have received the second dose of the vaccine (January 2022), of which AZD1222 accounts for 25% and BNT162b2 accounts for 55% (https://ncv.kdca.go.kr, accessed on 31 January 2022). 

Serious vaccine-related effects, including thrombotic thrombocytopenia, cerebral venous sinus thrombosis, splanchnic vein thrombosis, and pulmonary embolism, were reported after vaccination for COVID-19 [2,3,4,5,6]. In terms of ophthalmic reaction to COVID-19 vaccination, various manifestations including eyelid swelling, ptosis, superior ophthalmic vein thrombosis, acute graft rejection after keratoplasty, cranial nerve palsy, retinal vein occlusion, submacular hemorrhage, scleritis, uveitis, acute macular neuroretinopathy, optic neuritis, and paracentral acute middle maculopathy were found in several cases [7,8,9,10,11,12,13]. The COVID-19 vaccines with reported ophthalmic reactions included the mRNA vaccine (BNT162b2, Pfizer, Brooklyn, NY, USA; mRNA-1273, Moderna, Cambridge, MA, USA), vector vaccine (Ad26COVS1, Janssen Johnson & Johnson, New Brunswick, NJ, USA; AZD1222, Oxford–AstraZeneca, Cambridge, UK), and whole virus (PiCoVacc, Sinovac Biotech, Beijing, China; BBIBP-CorV, Sinopharm, Beijing, China) [14,15]. However, the causal relationship and the mechanisms by which these conditions develop remain unclear. Here, we present one of the largest reports of 17 cases with acute and severe ocular adverse events seemingly temporally related to COVID-19 vaccination that occurred following COVID-19 vaccination in a single center.

## 2. Materials and Methods

This single-center, retrospective observational case series adhered to the tenets of the Declaration of Helsinki and received approval from the Institutional Review Board of the Korea University Medical Center (IRB no. 2021GR0402). Given the retrospective nature of the study, the institutional review board of the Korea University Medical Center waived the need for informed patient consent. We included patients who experienced ophthalmic symptoms and were diagnosed with a new ophthalmic disease or exacerbation of a previously diagnosed ophthalmic disease following vaccination for COVID-19, from March 2021 to January 2022, at Korea University Guru Hospital in Seoul, Korea. Only patients in whom these symptoms occurred within seven days after inoculation were included. We followed the reporting guidelines for case-series studies [16] and noted the limitations of an uncontrolled design; however, we highlighted the importance of prompt reporting in the field of COVID-19 because of the ongoing pandemic and drastic increase in the number of vaccinations.

Information on the patients’ age, sex, medical and ophthalmic history, diagnosis, visual acuity, and treatment was obtained. The visual acuity is presented with the Snellen scale. The name, dose, and administration date of the vaccine were also obtained through detailed history-taking. All eyes with retinal disorder and uveitis underwent ultra-wide-field fundus photography using a fundus camera (Optos Inc., Dunfermline, UK) and spectral-domain optical coherence tomography (OCT; Spectralis OCT; Heidelberg Engineering, Heidelberg, Germany). Eyes with retinal vein or artery occlusion and posterior uveitis were subjected to fluorescein angiography (FA) using a Spectralis HRA+OCT device (Heidelberg Engineering, Heidelberg, Germany). Angle-closure glaucoma (ACG) patients underwent examinations including axial length (AL) (IOL Master, Carl Zeiss Meditec, Dublin, CA), and central corneal thickness (SP-2000P specular microscope Topcon Medical Systems, Tokyo, Japan) measurements along with intraocular pressure (IOP) measurement using Goldmann applanation tonometry, anterior segment optical coherence tomography (Cirrus HD-OCT; Carl Zeiss Meditec, Jena, Germany), and 24-2 Swedish interactive threshold algorithm standard automated perimetry (Carl Zeiss Meditec, Jena, Germany). Ultrasound biomicroscopy (Model P60, Paradigm Medical Industries Inc., Salt Lake City, UT, USA) was performed in only one ACG patient. Laboratory tests, including complete blood count, prothrombin time, activated partial thromboplastin time, and comprehensive metabolic profiles were performed in 11 of 17 patients. In RVO patients, an anti-PF4 antibody assay was performed for possible cases (*n* = 4). The mean and standard deviation (mean ± SD) of the clinical parameters are presented.

## 3. Results

Seventeen eyes of 16 patients were included in this study. Of the study participants, 12 patients (75%) had been vaccinated with the AstraZeneca vaccine (AZD1222, Cambridge, UK) and 4 (25%) with the Pfizer vaccine (BNT162b2, Pfizer, New York, NY, USA and BioNTech, Mainz, Germany). Table 1 summarizes the characteristics of the patients in this report. All patients in this case series were Asian. The mean age at symptom presentation was 63.8 ± 11.9 (range 33–83) years. RVO was most commonly observed (nine eyes, 52.9%); branch retinal artery occlusion was observed in one eye (5.9%). Among the three uveitis patients (17.6%), one eye had no history of uveitis but presented with anterior uveitis after vaccination; two eyes had a controlled panuveitis history and showed worsening of panuveitis after vaccination. In four eyes (23.5%), an angle-closure attack that did not improve with glaucoma eyedrops and laser iridotomy was observed. All patients visited our clinic complaining of decreased visual acuity and ocular pain was observed in ACG patients, and visual symptoms occurred at an average of 3.5 ± 2.3 (1–7) days after inoculation.

### 3.1. RVO (Cases 1–9)

**Figure 1:**
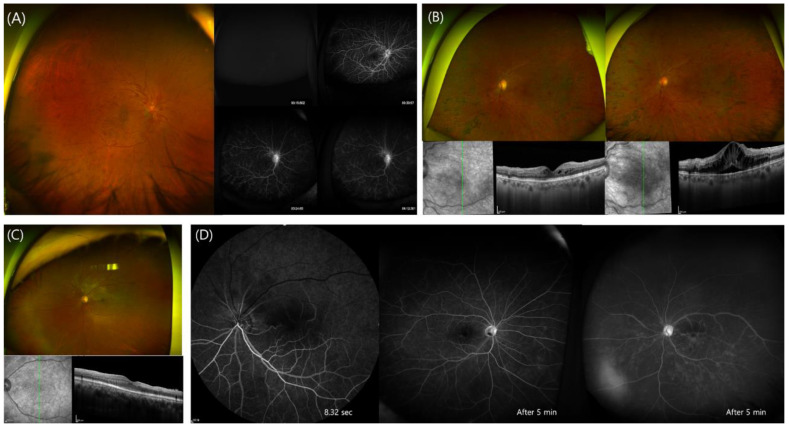
(**A**) (Patient 1) A 64-year-old man, who had no previously diagnosed disease, visited our clinic complaining of reduced visual acuity (VA) in the right eye one day after AZD1222 inoculation. (**Left**) Severe vessel tortuosity with scattered blot retinal hemorrhage was observed during a fundus examination of the right eye. (**Right upper**) Early phase (arterial phase) delay and arterio-venous transit time were found during fluorescein angiography, indicating central retinal vein occlusion. (**Right lower**) Multiple focal leaks and disc hyperemia in the late phase were also observed. (**B**) (Patient 4) A 63-year-old woman was diagnosed with central retinal vein occlusion in her left eye in January 2021 at our hospital and underwent pan-retinal photocoagulation of the left eye and four intravitreal anti-vascular endothelial growth-factor injections. (**Left**) At her last visit just before vaccination, the retinal hemorrhage in the left eye was hardly visible, vessel tortuosity was not severe, and a focal intraretinal cyst was observed on OCT images. Additionally, her VA was 20/360. (**Right**) The patient reported that three days after AZD1222 vaccination, her vision had deteriorated, and her visual acuity as measured at the outpatient clinic was 20/360. It was confirmed via fundus examination that the vessel tortuosity was also greatly increased, and there was macular edema present on OCT images. (**C**,**D**) (Patient 5) A 62-year-old man visited the hospital complaining that one-third of his visual field in the left eye was blurred (VA 20/63) one day after AZD1222 vaccination. (**C**) During fundus examination, retinal whitish ischemic changes at the superotemporal arcade were observed, and inner retinal swelling due to acute ischemia was confirmed on OCT images. (**D**) During the FA examination, a filling delay in the superior retinal arteries in the early phase was observed, and in the wide FA photograph, non-perfusion of the relevant area was observed in the late phase. Notably, both eyes had mild vascular leaks, suggesting vasculitis.

RVO was diagnosed in nine eyes of eight patients (three men and five women) by fundus examination and FA (Figure 1). The mean age at symptom presentation was 62.1 ± 13.4 years (range: 33–74 years). One patient who showed vitreous hemorrhage with branch RVO in both eyes had hypertension (12.5%), but other patients had no systemic disease. Six patients (75%) received the AZD1222 vaccine, and two (25%) received the BNT162b2 vaccine. Two patients with the Pfizer vaccine were 33 and 48 years old, respectively. Four patients (44.4%) experienced RVO after their first dose of vaccination, three (33.3%) after their second dose, and one (11.1%) after the third dose. Five patients (Cases # 1–5) showed newly developed RVO but demonstrated relatively preserved visual acuity (VA) (20/125 to 20/20), while four eyes of three patients (cases # 6–9) showed exacerbation of existing RVO and experienced significant vision loss due to dense vitreous hemorrhage and macular edema (VA hand motion to 20/630). The three eyes of cases #6–8 had a history of vitrectomy due to vitreous hemorrhage which accompanied the RVO. The mean time between vaccination and visual symptom development was 4.3 days (range: 1–7 days). Five eyes were treated with intravitreal bevacizumab injection (IVB), and one patient who showed vitreous hemorrhage in both eyes underwent vitrectomy because the vitreous hemorrhage was not resolved after IVB. The hematologic evaluation was performed in six patients, and anti-PF4 antibodies assay was performed in three patients, but no abnormal findings were observed.

### 3.2. Retinal Artery Occlusion (Case 10)

A 62-year-old man, who had received the first dose of the AZD1222 vaccine one day prior to presentation, complained of a central visual field defect and reduced VA (20/63). He had hypertension, diabetes, and a history of cerebral infarction in 2017. Fundus examination revealed retinal whitish ischemic changes at the super temporal arcade on OCT images (Figure 1C). A filling delay in the superior retinal arteries in the early phase was observed in the FA examination, and both eyes had mild vascular leaks in the late phase, suggesting vasculitis. (Figure 1D). No abnormal finding was observed in the hematologic evaluation. 

### 3.3. Uveitis (Cases 11–13) 

**Figure 2:**
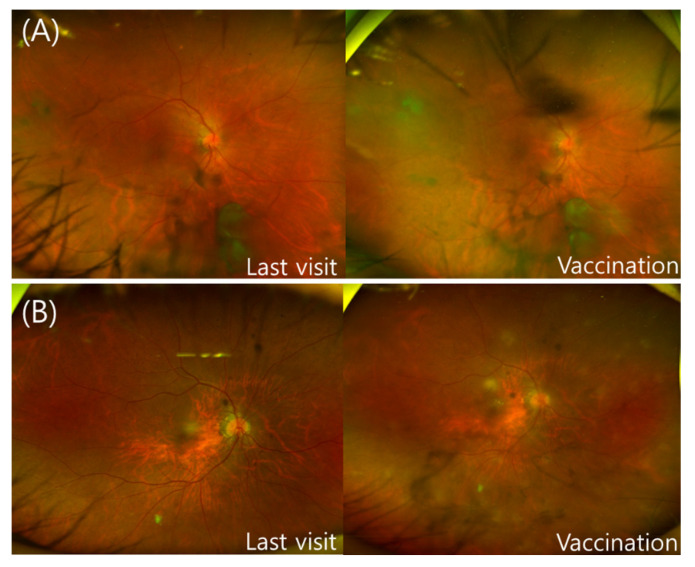
(**A**) (Patient 11) A 62-year-old man, previously diagnosed with panuveitis with human leukocyte antigen B51 positivity and who was taking an oral steroid and cyclosporin (**Left**), shows well-controlled uveitis, although vitreous opacity remains (visual acuity 20/40). (**Right**) The patient reported a decrease in visual acuity (VA) in the right eye one day after AZD1222 inoculation, documented as 20/100. An increase in vitreous opacity in the right eye was confirmed during fundus examination, and keratic precipitates and inflammatory cells in the anterior chamber during the slit-lamp examination were observed. (**B**) (Patient 12) The disease of a 79-year-old woman, who was diagnosed with panuveitis 20 years prior and undergoing follow-up, was well-controlled without topical and systemic medications (right eye VA 20/63). (**Left**) A relatively clear vitreous without signs of inflammation during fundus examination was observed at the last visit. (**Right**) The patient was inoculated with BNT162b2, and three days later, she complained of decreased VA in the right eye (20/200) and stiff pain in both eyes. During fundus examination, vitreous opacity and focal retinal infiltration were observed in her right eye.

Cases 11 and 12 were of patients with a history of panuveitis accompanied by vasculitis as an underlying disease (Figure 2A,B). Patient 11 was positive for human leukocyte antigen B51 at the time of diagnosis of panuveitis, and steroid and cyclosporine were orally administered, while patient 12 was in a stable state without the need for medication. These patients were inoculated with the AZD1222 vaccine and BNT162b2 vaccine, respectively, and both visited the clinic due to reduced VA after one and three days, respectively. In both cases, greater vitreous opacity, keratic precipitates, and an increase in the number of inflammatory cells in the anterior chamber were observed. Both cases were followed up until recently (May 2022). Case #11 received three intravitreal triamcinolone injections for macular edema and phacoemulsification for the cataract. PO cyclosporin and methotrexate were maintained. The patient showed well-controlled inflammation with a final visual acuity of 20/40. Case #12 received retrobulbar triamcinolone injections twice for macular edema. Moreover, PO methotrexate was added to control inflammation. Her final visual acuity was 20/60. Case 13 was of a 55-year-old woman, who had hypertension and a history of branch RVO (in 2019) without other ophthalmic complications. The woman presented with anterior uveitis showing keratic precipitate and inflammatory cells in the anterior chamber two days after the third dose of the BNT162b2 vaccine. In FA, there were no signs of vasculitis or retinal or choroidal inflammation. The anterior uveitis was well controlled by topical steroids without recurrence. The hematologic evaluation in three patients showed no abnormal finding.

### 3.4. Primary Angle-Closure (Cases 14–17)

**Figure 3:**
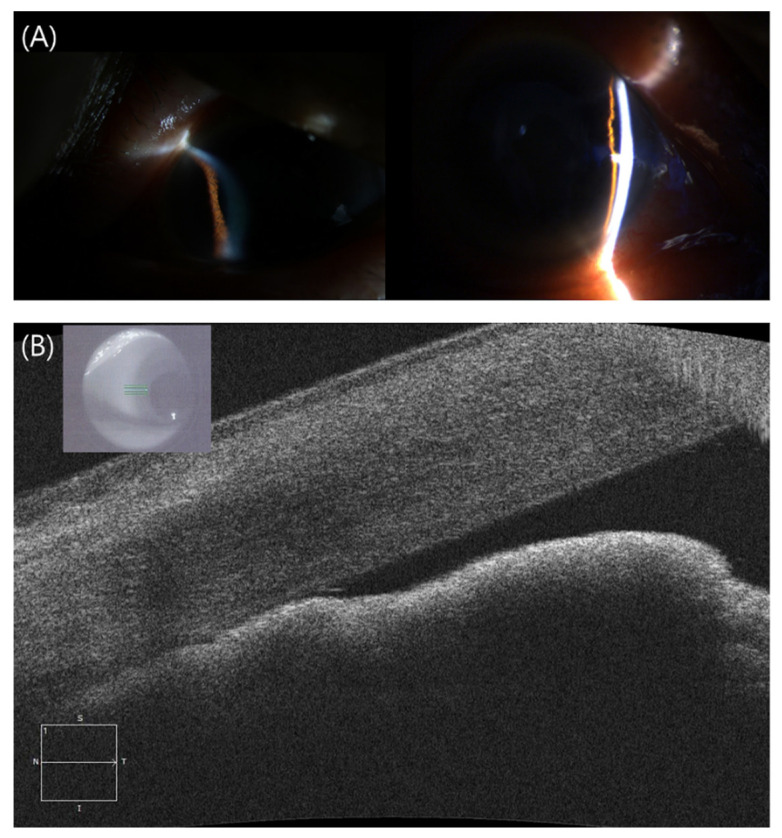
(Patient 14) A 71-year-old woman with hypertension presented to the emergency department with a two-day history of pain and redness in the left eye (visual acuity 20/50). (**A**) An ophthalmic examination revealed a high IOP of 36 mmHg associated with shallowing of the anterior chamber peripherally in the left eye. (**B**) Anterior-segment OCT images show anterior bowing of the peripheral iris and closing of the iridocorneal angle in the left eye. Her axial length was 21.86 mm in the right eye and 21.84 mm in the left eye, and her spherical equivalents were −1.13 and −3.63 diopters, respectively. An acute attack of angle closure was diagnosed, and treatment with laser peripheral iridotomy was attempted but failed. The next day, phacoemulsification with goniosynechialysis was performed and her IOP dropped to 7 mmHg.

Four patients visited the clinic following AZD1222 vaccination with ocular pain and significant acute visual loss, displaying corneal microscopic cystic edema with conjunctival injection, a shallow central anterior chamber, and peripheral anterior chamber collapse nearly touching the cornea with a phakic eye (Figure 3A,B). Our four subjects’ anterior chamber depths (ACDs) were 2.19 mm, 2.19 mm, 2.89 mm, and 2.31 mm, respectively. Their axial lengths were 21.84 mm, 22.95 mm, 22.37 mm, and 23.71 mm, respectively. All four patients presented high IOP values (36, 66, 70, and 34 mmHg) and traced to one positive anterior chamber cell reaction in attacked eyes. Their spherical equivalents (SE, right eye/left eye) were −1.1/−3.63 diopters, −2.6/−3.75 D, −2.25/+1.15 D, and +0.5/+2.86 on the initial visit day after an attack, respectively. Case #16 had visited our clinic 2 weeks before the ACG attack for meibomian gland dysfunction. Hence, we were able to compare her refraction at the time of the ACG attack relative to the previous record. The SE of her right eye was −0.89 diopters at 2 weeks before the ACG attack and −2.25 diopters on the day of the ACG attack. The patient demonstrated myopic shift from her prior measurement. For IOP control, case #14 underwent phacoemulsification with goniosynechiolysis because her gonioscopic exam represented a 360° peripheral anterior synechia (PAS). Case #15 underwent trabeculectomy with Argon laser peripheral iridoplasty. The laser iridotomy did not achieve satisfactory IOL lowering effects. Case #17 had phacoemulsification with posterior chamber lens implantation. In Case #18, we decided to perform a vitrectomy with IOL scleral fixation, as his lens showed anterior shift with phacodonesis due to zonule laxity. All four cases showed generally good prognosis in terms of intraocular pressure and final visual acuity (20/20, 20/100, 20/20, and 20/25). All patients were free of corneal complications except case #15, who demonstrated a decreased corneal endothelial cell count before trabeculectomy. The patient recovered a clear cornea after surgery without edema. 

## 4. Discussion

Herein, we report a case series of acute ocular adverse events related to COVID-19 vaccination. Our ophthalmic clinic is a tertiary hospital located in the Guro district in Seoul. We care for approximately 60,000 people in the outpatient department annually. Among a total of 393,822 people in Guro district, 333,958 have received the first vaccination dose and 43,214 have received the second vaccination dose, based on our community’s Public Health Service announcement). Our clinic is a tertiary hospital. Patients from other districts are referred to us. Hence, determining the prevalence was challenging. As this was a retrospective study analyzing patient medical records, there were certain limitations to definitively confirming a causal relationship between such immunization and the noted ocular manifestations. Furthermore, laboratory and anti-PF4 antibody tests were performed only in some patients, and those who were tested showed negative results. Laboratory tests cannot prove a correlation between ocular adverse events and the vaccination; however, we only included patients who showed acute ocular symptoms within one week of vaccination to determine the temporal association of COVID-19 vaccination and ocular adverse events.

In this study, we presented a large case-series of ocular adverse events after COVID-19 vaccination, including nine RVO cases, one retinal artery occlusion case, three uveitis cases, and four cases of acute angle closure, which has not been reported previously. In the cases of RVO, which was the most common ophthalmic manifestation in this report, only one of eight patients (12.5%) had hypertension, which was much lower than that in a previous report of RVO cases among Koreans (48.2%) [17]. The cases with vitreous hemorrhage (cases #6~8) had a history of vitrectomy due to vitreous hemorrhage which accompanied the RVO, meaning that there was previous neovascularization. Park et al. reported on submacular hemorrhage and vitreous hemorrhage in a patient with age-related macular degeneration and RVO [7]. The vessel vulnerable to microvascular dysfunction including neovascularization due to AMD and RVO may develop hemorrhagic complications after COVID-19 vaccination. 

The AZD1222 vaccine is an adenovirus vector vaccine containing the coding region for the severe acute respiratory coronavirus disease 2 (SARS-CoV-2) spike protein gene, which triggers a strong innate inflammatory response [18]. Conversely, the BNT162b2 is a lipid nanoparticle formulated nucleoside-modified RNA encoding the SARS-CoV-2 full-length spike protein [19]. Spike proteins produced by adenovirus-vectored vaccination mimic the SARS-CoV-2 spike protein’s receptor binding structure [20]. SARS-CoV-2 uses the spike protein to invade cells by attaching to angiotensin-converting enzyme 2 receptor (ACE2) as the target receptor. The interactions between ACE2 and free-floating spike proteins enhance the overactivity of angiotensin II, which may help to trigger inflammation, thrombosis, and other adverse reactions [18]. A previous analysis of fundus photography of patients with COVID-19 revealed increased artery and vein diameters and tortuosity [21], while another OCT angiography analysis documented reduced vessel density with and without thromboembolism [22], supporting the findings of retinal vascular inflammation in COVID-19 patients. Although there are no reports on the effects of COVID-19 vaccines on the retinal artery and vein, inflammation in the retinal vessels caused by adenoviral vector vaccines and spike proteins can be predicted along with vein compression or microthromboembolic events due to increased vessel tortuosity and arterial vessel dilation. 

Vaccination can induce all types of uveitis, albeit mainly transient anterior uveitis and sometimes vasculitis, as well as panuveitis [23]. The incidence of uveitis after general vaccination was reported to range from 8 to 13 per 100,000 persons/year [24]. Mudie et al. assumed some possible causes of this rare type of uveitis [10]. One is molecular mimicry between the vaccine and ocular structures driving the adaptive immune system to induce autoimmunity. The self-reactive immune system may cause uveitis as well [25]. Another hypothesis is that an enhancement of the systemic innate immune system results in significant cytokine activation. The exacerbation and new development of uveitis in patients 11 and 12 might be due to innate and adaptive immune reactions to the vaccine, although molecular mimicry between the vaccine and ocular structure is another possibility [10]. 

**Figure 4:**
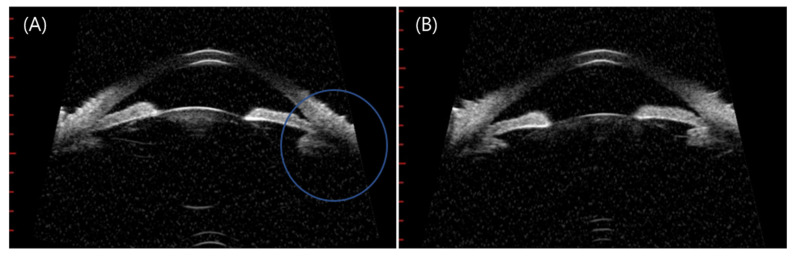
(Patient 17) (**A**) Right eye of a 64-year-old man, who had no previously diagnosed disease. He visited our clinic complaining of pain and redness in the right eye 6 days after AZD1222 inoculation. (**B**) His left eye shows a normal peripheral angle and no zonule laxity. His initial IOP was 34 mmHg associated with shallowing of the anterior chamber peripherally in the right eye. His bio-microscopy images show anterior bowing of the peripheral iris and ciliary swelling in the right eye than the left eye, which caused phacodonesis of the right eye and closure of the iridocorneal angle in the right eye. An acute attack of angle closure was diagnosed, treatment with laser peripheral iridotomy was attempted, and his IOP dropped to 10 mmHg.

Further, we hypothesized the main etiology of the four angle-closure attack cases following COVID-19 vaccination is ciliary body swelling due to uveitis. Not all patients were photographed, and Ultrasound biomicroscopy figures were available in one case in our study. Figure 4 shows swelling of the ciliary body six days after vaccination that led to zonule laxity accompanied by phacodonesis, causing a closed-angle attack. The swollen ciliary body may lead to anterior shifting of the lens and, consequently, to a myopic shift. Although we only compared SE before and after the ACG attack in case #16, the other cases were more likely to have myopic SE in the affected eyes than the fellow eye. The median age of our four ACG patients was 69.5 years, and the women to men ratio was 3:1, similar to that in a multi-centered Korean study with an average age of 64.28 years and sex ratio of 3.13:1 [26]. Axial lengths of our ACG subjects were 21.84 mm, 22.95 mm, 22.37 mm, and 23.71 mm, which were similar with average 22.42 mm in one of acute primary angle-closure studies in Korea [27]. Conversely, our subjects’ anterior chamber depths (ACDs) were 2.19 mm, 2.19 mm, 2.89 mm, and 2.31 mm, which were deeper than the average ACDs in ACG cases in Korea (1.87 mm) in 2017 [27]. 

Moreover, hypersecretion of aqueous humor may be a contributing factor. The renin-angiotensin system has been identified in the human ciliary body and aqueous humor, and angiotensin II acts as a secretagogue in human ciliary non-pigmented epithelial cells [28]. The loss of ACE2 due to interactions between spike proteins produced by COVID-19 vaccination may lead to the overactivity of angiotensin II [18] and its increase in the aqueous humor. Even considering the high prevalence of ACG in Asia, this mechanism would be worth considering when treating patients with ACG following COVID-19 vaccination [29]. 

AZD1222 and BNT162b2 were administered to different populations in accordance with government guidelines. AZD1222 was mainly inoculated in older individuals, and the age limit was changed from over 30 years to over 50 years during the study period. However, BNT162b2 vaccine was inoculated in individuals over 16 years of age without an upper age limit. Therefore, it is impossible to compare adverse events between the two vaccines, and it is considered that relatively more ocular adverse events were reported with AZD1222, which had an older inoculation age. Some of the patients included in this report had an underlying disease, and although the possibility of coincidental ophthalmic manifestation due to the underlying disease cannot be excluded, since a large number of cases of ocular adverse events were noted in a single-center study in a limited period, a temporal association between vaccination for COVID-19 and ophthalmic disorders is more likely.

Ocular manifestations in patients infected with COVID-19 have been reported. Most were mild, such as conjunctival congestion, conjunctivitis, dry eye, and keratitis [30]. Case reports of retinal and choroidal manifestations after COVID-19 injection have highlighted retinal microvascular changes including cotton wool spot, intraretinal hemorrhages, paracentral acute middle maculopathy, acute macular neuroretinopathy, retinal vein occlusion, and uveitis [31]. Although the mechanism of these ocular manifestations has not been clearly elucidated, the presence of SARS-CoV-2 in the vitreous and retina of patients with COVID-19 [32] may implicate the viral infection directly or contribute to immune-mediated inflammation. The ophthalmic complications after COVID-19 infection are similar to the adverse events after COVID-19 vaccination. 

This was a single-center retrospective review, and no incidence analysis was performed. In addition, there is no appropriate control group, and since it was not a survey of patients who had been vaccinated in a single center, only observational reports could be included. This study only included severe ocular adverse events that affected visual acuity. Other ophthalmic events, such as eyelid swelling, ptosis, and cranial nerve palsy that were previously reported may not have been considered. Furthermore, systemic evaluations were not conducted uniformly in all cases. Population-based studies with national health insurance records for the evaluation of the incidence of ocular adverse events before and after COVID-19 vaccination may help determine the correlation.

Because of the absence of a prevalence analysis, the causality and direct correlation between ocular reactions and COVID-19 vaccination cannot be determined from this report. However, the possibility of a temporal association between the reported ophthalmic manifestations and COVID-19 vaccination provides a new perspective. Although prevalence assessment was not conducted, the strength of our study was its homogeneity, as only Asians were included. The reported ophthalmic events were unexpected. Ocular adverse events are relatively rare, and the benefits of vaccination outweigh the risks if appropriate ophthalmic management is followed. Adopting an enhanced watchfulness protocol, especially for the at-risk patients—including recipients of corneal grafts, and those with a history of uveitis and retinal vascular disease—may be necessary after COVID-19 vaccination.

## 5. Conclusions

Clinicians should consider the effect of COVID-19 vaccination on ophthalmic disease. Further studies are required to elucidate the possible effects of COVID-19 vaccination on the eye.

## Figures and Tables

**Table 1:** Case presentations.

Case	Age	Sex	Vaccination	Dose of Vaccination	Laterality	Symptom Onset ^a^	VA (Snellen)	VA before Vaccination (If Known)	Diagnosis	Systemic Underlying Disease	Ophthalmic Underlying Disease (Year of Diagnosis)	Previous Medication (s)	Treatment
1	64	M	AZD1222	1	RE	1	20/25		CRVO	None	None	None	Observation with aspirin
2	33	F	BNT162b2	2	RE	6	20/40		CRVO	None	None	None	Anti-VEGF injection
3	48	M	BNT162b2	3	RE	6	20/125		CRVO	None	None	None	Anti-VEGF injection
4	69	F	AZD1222	1	LE	3	20/20		BRVO	None	None	None	Observation with aspirin
5	66	M	AZD1222	2	LE	7	20/20	20/20	BRVO	None	None	None	Observation
6	68	F	AZD1222	1	RE	1	Hand motion		BRVO with vitreous hemorrhage	None	BRVO (2020)	Aspirin	Observation
7	74	F	AZD1222	2	RE	6	Hand motion	20/25	BRVO with vitreous hemorrhage	HTN, Nasal cavity cancer (CTx. Complete remission–2016’)	BRVO (2020)	Aspirin	Vitrectomy
8 (the other eye of case #7)	74	F	AZD1222	2	RE	6	Hand motion	Hand motion	BRVO with vitreous hemorrhage	HTN, Nasal cavity cancer (CTx. Complete remission–2016’)	BRVO (2020)	Aspirin	Vitrectomy
9	63	F	AZD1222	1	LE	3	20/630	20/630	CRVO decompensation	None	CRVO (2021)	None	Anti-VEGF injection
10	62	M	AZD1222	1	LE	1	20/63	20/25	BRAO	HTN, DM, Cerebral infarction (2017’)	ERM Secondary glaucoma	Clopidogrel	Observation
11	62	M	AZD1222	1	RE	1	20/100	20/40	Uveitis exacerbation	HTN	Controlled panuveitis	PO steroid PO cyclosporine	Steroid ^b^ PO methotrexate
12	79	F	BNT162b2	1	RE	3	20/200	20/63	Uveitis exacerbation	DM, Asthma	Controlled panuveitis	None	Steroid ^b^ PO methotrexate
13	55	F	BNT162b2	3	RE	2	20/50	20/25	Anterior uveitis	HTN	BRVO (2019)	None	Steroid ^b^
14	71	F	AZD1222	1	LE	6	20/50		ACG attack	HTN	None	None	Phaco with goniosynechiolysis
15	83	F	AZD1222	1	RE	3	Counting finger		ACG attack	None	None	None	Trabeculectomy ^c^
16	59	F	AZD1222	1	RE	1	20/100	20/25	ACG attack	HTN	ACG (2021)	Dorzolamide and timolol eyedrop	Phaco ^d^
17	64	M	AZD1222	2	RE	6	20/50		ACG with Lens displacement	None	None	None	Vitrectomy

^a^ no. of days after vaccination. ^b^ Topical steroid, retrobulbar triamcinolone injection and per oral steroid. ^c^ Laser iridotomy and argon laser peripheral iridoplasty were failed to control IOP. ^d^ Laser iridotomy was tried but failed to control IOP. Abbreviations: RE, right eye; LE, left eye; VA, visual acuity; CRVO, central retinal vein occlusion; BRVO, branch retinal vein occlusion; BRAO, branch retinal artery occlusion; ERM, epiretinal membrane; ACG, angle-closure glaucoma; HTN, hypertension; DM, diabetes mellitus; PO, per oral; VEGF, vascular endothelial growth factor; Phaco, phacoemulsification.

## Data Availability

The datasets used and analyzed during the current study are available from the corresponding author on reasonable request.
